# Living the best of both worlds: A personal scientific journey

**DOI:** 10.1096/fba.2021-00103

**Published:** 2021-11-05

**Authors:** Gregory J. Tsongalis

**Affiliations:** ^1^ Dartmouth Hitchcock Medical Center Lebanon, NH and Geisel School of Medicine at Dartmouth Hanover New Hampshire USA

**Keywords:** clinical genomics, Laboratory Medicine, molecular pathology

## Abstract

Opportunity is the essence of a career in science or medicine and this continues to be a major source of satisfaction for the many scientists and health care providers worldwide. Having trained as a PhD scientist, the world of clinical medicine seemed to be a galaxy away. I could not have been more wrong in that assumption. Here, I tell the story of my career trajectory so that those new to the sciences understand the potential and the opportunities afforded by a career in clinical laboratory medicine.

## 1 INTRODUCTION

Many types of careers have defined tracks, those paths that individuals follow, that lead to scaling career ladders and successful careers. In science and medicine, traditional career tracks have been historically well‐defined with some individuals choosing to pursue basic or translational research while others choose to focus on patient care. What has become grossly obvious is the intersection of these two paths as the science of medicine becomes more complex and less understood. Often, we hear of the physician/scientist track whereby practicing providers also partake in some type of research effort. Rarely do we hear of the scientist partaking in patient care efforts. That is the path that my career inadvertently followed and it has allowed me to enjoy the satisfaction of indirectly providing patient care while also partaking in translational research during the era of genomic medicine.

## 2 THE ROAD LESS TRAVELED

The surgical orderly entered the pathology gross lab carrying a large black trash bag containing a piece of human tissue a bit larger than a basketball. I was a graduate student in a Masters of Health Science program to train as a Pathologist Assistant (PA) at Quinnipiac College, this was my rotation at a local hospital in Connecticut, and the year was 1985. I had completed my BS degree in zoology with a minor in chemistry from the University of Massachusetts at Amherst and was now in my second year of this PA program where we rotated thru various hospitals to perform autopsies and process surgical specimens.

As students, we were immersed in human disease, seeing firsthand the consequences of myocardial infarction, diabetes, infectious agents and cancer. At that time, a new infectious disease had emerged that seemed to be lethal in the homosexual community by rendering infected individuals susceptible to other life‐taking pathogens. Both research and clinical laboratories were involved in the discovery of the virus causing acquired immunodeficiency syndrome (AIDS) and the other pathogens that would kill millions of infected people worldwide. This was my first exposure to the types of data and the impact clinical laboratories could have on patient care. The title of my Master‘s thesis (typed manually for each edited version) was “A Retrospective Study of *Pneumocystis carinii* by Fluorescent Microscopy in Papanicolaou Stained Pulmonary Specimens.” Not only was this a timely study trying to identify a more rapid method to detect *P. carinii*, as this organism was one of the main causes of mortality in AIDS patients, it was my first true exposure to translational research.

While the didactic portion of the PA curriculum taught us about gross and microscopic pathology procedures, dissection techniques and pattern recognition, very little was discussed at the time about the underlying etiology of the diseases we were examining. I recall going to the West Haven VA Hospital library to do manual literature searches on various diseases, and every time I searched on a different tumor type, the literature was flooded with new molecular biology tools and findings of genes thought to be causative of cancer. P53, KRAS, MYC were just a few of the gene names that would dominate the cancer literature over the next few years. The link between mutation, altered pathway functions and prognosis was being made right before my eyes as the science moved ahead at a whirlwind pace. My interest was clearly kindled and, wanting to learn more about these molecular phenomena, I applied to various PhD programs.

Upon completing the PA program, I entered a PhD program in the Department of Pathology at the University of Medicine and Dentistry of New Jersey (currently Rutgers University) where I would learn much more about human disease and their underlying mechanisms. After several courses and lab rotations with prominent faculty such as Dr. George Studzinski and the late Dr. Nicholas Ponzio, I was accepted into the laboratory of Drs. Clark and Muriel Lambert. Here I would study DNA repair mechanisms, learning the basics of cell culture, protein analysis, electroporation, in situ hybridization and the processes of the scientific method that are used routinely in any investigation.^1^, ^2^, ^3^ My doctoral dissertation was titled “The Ability of Normal Human DNA Endonucleases to Complement the DNA Repair Defects in Xeroderma Pigmentosum and Fanconi‘s Anemia,” it was 1990.

Absolutely amazed by the biological and environmental risk factors that could cause cancer, I sought out post‐doctoral fellowship programs that would allow me to build a career as a cancer molecular biologist and expand on what I had already learned. This path took me to the Department of Pathology at the University of North Carolina at Chapel Hill and into the laboratory of Dr. David Kaufman. I was introduced to new and creative thought processes, new techniques and new concepts that would impact one of the most fundamental characteristics of human cancer, DNA replication and its association with the nuclear matrix.^4^, ^5^ I mentioned “new” three times in the previous sentence because this was important for my growth both academically and personally. Many times students settle into a comfort zone in a lab or institution and never leave, but as PhD scientists we are trained to think outside of the box and approach challenges from different perspectives gained from mentors we have had during our training. Dr. Kaufman provided an atmosphere of creative and free thinking while focusing on complex scientific questions. It was here that I met long‐time colleague, collaborator and friend, Dr. William Coleman, who was doing a post‐doc with Dr. Joe Grisham in the same department. At the time, Bill and I would collaborate on smaller side projects as time allowed, and we were the first to publish on the use of Alu PCR for the identification of human biological samples.^10^

It was towards the end of my 2‐year fellowship in the Kaufman lab that I was asked the question, “So what are you going to do next?” I happened to be sharing an office with one of the pathology residents (the late Dr. Anne Kellogg) who was doing some research in the Kaufman lab as well. I responded that I had been looking for another post‐doc on the west coast but really wanted to do something a bit more clinically oriented and use the experience I had gained as a PA. Anne stood up from her desk, took me by the hand and walked me over to the adjacent building which housed the UNC Hospitals Clinical Laboratories. She introduced me to Dr. Robert Cross who was the director of the Clinical Chemistry post‐doctoral training program. I explained that I was not a chemist but a molecular biologist after which Dr. Cross mentioned they had recently introduced molecular diagnostics into their training program. He introduced me to Dr. John Chapman (Director of the Clinical Chemistry Laboratory), Dr. Donald Forman (clinical chemist), Dr. John Hammond (clinical chemist and lab information systems) and Dr. Larry Silverman who oversaw the new molecular diagnostics lab. All were PhD trained scientists directing a clinical laboratory. Dr. Silverman provided rigorous training in clinical diagnostics and supported the exploration and development of new clinical tests and technologies in molecular pathology.^6^, ^7^, ^8^ I spent the next 2 years learning about clinical laboratory operations, diagnostic testing and biomarkers of disease and the all too dreadful regulatory aspects of a clinical laboratory.

We were required to become very familiar with the College of American Pathologist‘s checklists for laboratory inspections, keep pace with all of the new developments in human genetics, stay abreast of the technology as it changed from Southern blot transfer analysis to the polymerase chain reaction and fully validate one new test during our 2‐year fellowship. At the time, there were no molecular diagnostic tests for oncology and molecular infectious disease testing did not exist. I validated a Southern blot procedure for fragile X syndrome using a radioactively labeled probe.^9^ During this fellowship, I recognized the potential that molecular diagnostics could have for clinical applications that could only be fulfilled if we had a robust research approach to evaluate new technologies as they were developed and new genomic biomarkers as they were discovered. I am forever grateful to Anne for that question and that walk and to Larry for the many years of mentorship.

Upon completion of my fellowship, I was hired by Dr. Alan Wu to join him and Drs. Robert Moore and Robert Burnett in the Clinical Chemistry Laboratory, Department of Pathology at Hartford Hospital. Once again all were PhD trained scientist directing a clinical laboratory. The charge to me was to develop a molecular diagnostics service that would provide state‐of‐the‐art molecular testing to the patient population of this 1200 bed inner city hospital. Dr. Wu provided an incredible atmosphere of collegiality, collaboration, mentorship and friendship. After 10 years at Hartford Hospital where I was employed by Hartford Pathology Associates, a private practice pathology group, the atmosphere at the institution changed from academic to business and I found myself looking for employment elsewhere. It so happened that the director of the molecular genetic laboratory at Dartmouth was retiring, and that opportunity landed on my doorstep. In 18 years at the Dartmouth Hitchcock Medical Center, I was able to grow the lab from three full‐time equivalents (FTEs) to over 50 FTEs and expand clinical genomic testing to span genetics, infectious diseases and oncology applications. I am currently the Director of Clinical Genomics and Advanced Technology (CGAT) and am the Vice Chair for Research in the Department of Pathology and Laboratory Medicine for the Dartmouth Hitchcock Health System.

## 3 THE TECHNOLOGY REVOLUTION

The journey that my career has taken was heavily impacted by the changing technologies that allowed us to explore and exploit the genome to extents never possible. In the blink of an eye, it seemed like the entire genomic revolution was upon us. New techniques, new diseases and new molecular markers for those diseases were expanding at a pace that was difficult to keep up with, to say the least. There were two main drivers in the field of precision or genomic medicine. First were the new targeted therapeutics that almost always mandate knowledge of the patient‘s genome before an appropriate therapy could be selected, the so called companion diagnostic. Second were the technological advancements that allowed us to ask questions we were never able to ask. At the same time that all of these new scientific, medical, and technical advances were being introduced into the clinical laboratory and healthcare practices, we also had to implement them within the constraints of the hospital lab‘s overwhelming and growing regulatory environment. There was no room for lowering quality standards when it came to test results that impacted patient care, and new metrices for quality assurance were born with each new technological advancement. Operationally, parameters such as cost, equipment, staff and turnaround time (TAT) for reporting of results consumed much time and resources.

When developing a Southern blot transfer test for fragile X syndrome, it didn‘t take long to understand that there were few ways to improve the TAT of this method. Isolating DNA, restriction digest, electrophoresis and transfer, probe hybridization, washes, and detection could take an excess of two weeks if all went well. Chemiluminescent detection systems helped to improve the TAT somewhat but you still needed fresh tissue or blood to isolate high molecular weight DNA which was a major limitation for pathology labs harboring hundreds of thousands of formalin‐fixed, paraffin‐embedded (FFPE) tissue blocks in their archives. While other applications were being quickly developed using a variety of blotting techniques for tests such as the gene rearrangements in B‐ and T‐cell lymphomas, it was apparent that new techniques were needed.

In 1985, a method first described by Kary Mullis allowed for the amplification of smaller pieces of DNA of interest that could then be analyzed using a variety of downstream techniques such as restriction enzyme digests, gel electrophoresis, probe hybridization, and Sanger sequencing. This method, known as the polymerase chain reaction (PCR) would revolutionize the use of molecular biology for diagnostic purposes and gain Kary the Nobel Prize. No longer were labs limited by specimen type and the many archived FFPE tissues were now fair game for analysis using this new method. In addition, the PCR allowed for many new clinical applications to be developed for genetic disease testing, infectious disease testing and oncology testing. Initially these tests could be completed in approximately 2–3 days, a vast improvement over the Southern blot assays, and with the development of and improvement to thermal cyclers the TAT could be reduced to within 1 day.

The PCR would allow us the flexibility to design and validate our own assays as quickly as we could dream them up. Many vendors had not yet begun to provide kits for different tests as a “market” had not yet been established. In retrospect, simple tests for detecting single point mutations in the Factor V, prothrombin (Factor II) and methylene‐tetrahydrofolate genes would provide the proof of principle that laboratories had the expertise to develop the then called “homebrew” or current laboratory developed test (LDT). We published an early technical paper on the ability to multiplex PCR amplification for the Factor V Leiden and the Factor II (prothrombin) gene mutations while also combining restriction enzymes for the detection of these two mutations in Connecticut Medicine.^11^ While this was not the prestigious scientific journal one aspires to publish in, it was the journal of the Connecticut State Medical Society with a large clinical readership in the state, allowing us to publish our findings while simultaneously advertising the presence and capabilities of our new lab. Germline tests seemed to be pretty straight forward with regards to primer design and detection method. It was not until we entered the realm of infectious disease testing that we learned how critical the nuances of specimen type and nucleic acid extraction methods would be on the sensitivity and specificity of the test.

With test menus growing in the lab, the new bottleneck was not in the analysis of the samples but instead it was with nucleic acid extraction. Labs would make their own extraction reagents and use either spooling or centrifugation steps to collect nucleic acid which was then re‐suspended in a working buffer solution. Industry recognized that robotics could solve the liquid handling steps of most extraction methods and allow for a much higher throughput of samples, in a shorter amount of time, with less labor. This development led to increases in testing volumes and to the expansion of test menus.

The field had been struggling with the limitations of post‐PCR detection methods, and in the mid‐1990s a modification to the PCR would be introduced that would once again revolutionize molecular testing in a clinical laboratory setting. That modification involved the introduction of dyes either directly into or attached as labels to primers or probes into the PCR. Thermal cyclers were also modified to be able to detect the fluorescent signal and the era of real time PCR was born. This change in chemistry and technology took PCR testing to another level as we could now rapidly detect sequences of interest and have very accurate quantitative testing capabilities. With additional automation and the need for more quantitative assays for viral load testing, molecular testing was becoming a routine part of the clinical laboratory. Additional modifications to real time PCR included multiplexing capabilities, more automation and the development of droplet digital PCR where each reaction vessel could contain up to 20,000 oil droplets, each harboring its own PCR reaction.

PCR was not the only molecular technology introduced to the clinical laboratory. In 1977, Walter Gilbert and Frederick Sanger developed two methods to determine the actual base sequence of DNA. They would share the 1980 Nobel Prize. The chain termination method developed by Sanger would become the method of choice for many laboratories and with the introduction of big dye terminators as well as capillary electrophoresis instruments would be the method used to sequence the first draft of the human genome. As with the evolution of PCR, there was a need for faster and more robust sequencing methods. Once again, changes to chemistry and instrumentation led to the introduction of massively parallel sequencing. This technique allows for the simultaneous sequencing of millions of fragments of DNA, spanning multiple genes or the entire genome from multiple patient samples. It has led to a precision medicine effort in oncology that is unprecedented, has allowed us to routinely sequence pathogen genomes, and has expanded inherited disease testing to whole exome and whole‐genome sequencing routinely.

I’ve given you a brief overview of the technological advances that have occurred during the course of my career. Each one of these advances led to numerous studies to evaluate the technology‘s performance and to apply it to a clinical question that may have gone unanswered by previous techniques. Often times we had novel ideas that could improve the technique for clinical use or address a simple or more complex question about human disease. Molecular diagnostics was a highly technical field where we needed to understand the nuances of how the assays and instruments would work in different situations. As these techniques become more complex and highly automated, the field of molecular diagnostics is playing more of a data informatics role as we generate terabytes of information from each test run. While the techniques are more complex, much of the bench work is being done by robotic instrumentation, allowing us to focus on questions and data interpretation.

## 4 CLINICAL ASSAY DEVELOPMENT

As my career in clinical genomics began, there were few clinical tests that were accepted but as time and technology progressed new DNA and RNA based tests were being developed at record speeds for both common and rare disease conditions. In the early days of assay development for molecular testing there were no guidelines or regulations on how to validate a new test. The birth of the Association for Molecular Pathology (AMP) in 1995 provided the infrastructure for formalizing standards of practice in molecular pathology and has become the premier organization for the discipline. There are few FDA approved in vitro diagnostic tests in the field today and most are for higher volume infectious disease testing. Our current clinical test menu includes more than 60 different tests of which only 13 are FDA approved. Laboratories are allowed to develop their own tests as long as proper validation steps are performed prior to using the test for clinical purposes to ensure robust performance with regards to sensitivity, specify, accuracy, and precision.

We developed and validated new assays for the detection of nucleic acid variants and targets associated with hereditary conditions, cancer and infectious diseases that were of both clinical and research interest.^12^, ^13^, ^14^, ^15^, ^16^, ^17^, ^18^, ^19^, ^20^ As the field grew and more disease genes identified, the laboratory validated the order of 8–12 new tests per year while also supporting research projects for students, residents, and fellows. Around the time of the O.J. Simpson trial a question of specimen identification arose in our department and I was asked to see if we could perform DNA testing on fixed tissue for identification purposes.^21^ This one case led to us developing an entire series of publications using molecular techniques to identify the origin of clinical specimens, especially those urine samples used in pre‐employment and employment drug screening programs.^22^, ^23^, ^24^, ^25^ There seemed to always be room for the development of new applications that could only be addressed by using molecular techniques.

There was no medical discipline that went untouched by molecular diagnostics and what we were able to develop was only limited by our own imagination. As equipment and techniques were refined, laboratories were able to develop and offer increasing numbers of clinical tests that could help serve the patient population at their institution. High throughput qualitative infectious disease testing began with the introduction of the Abbott LCX system for detecting *Chlamydia trachomatis* and *Neisseria gonorrhoeae* as a routine part of women‘s health screening programs. Quantitative PCR testing for infectious diseases introduced the concept of the viral load, our ability to detect a dynamic range of viral concentrations in patient plasma for HIV‐1 and HCV. Testing became available for genetic diseases such as cystic fibrosis, fragile X syndrome, and Duchenne muscular dystrophy. The number of genes and associated diseases we could test for was further exacerbated by completion of the human genome project.

In the area of human cancer, many tests were slow to develop in part due to the lack of cancer‐specific biomarkers and due to the question of actionability. HER2 became a target of interest in breast and other cancer types due to its impact on drug response, not for diagnosis. Our knowledge‐base of human cancer would significantly increase and impact diagnosis, prognosis and therapeutic selection. To date, many human cancers are now sequenced routinely using next generation or massively paralleled sequencing technologies to provide comprehensive genomic profiles. We introduced this technology to our clinical services using a 50 gene hotspot panel to identify variants in multiple genes that could be actionable.^26^, ^27^, ^28^, ^29^, ^30^ Currently, we sequence 170 genes and are now moving forward with whole‐exome sequencing for somatic and germline variant detection. Sequencing and microarray technologies continue to evolve and dominate the diagnostics field along with new modifications to real time and digital PCR techniques. A shift to higher complexity techniques, more automation and informatics capabilities are being counteracted by easy to use plug and play systems that can be performed in smaller hospital laboratories, clinics and near patients. I find the impact that my laboratory has on daily patient care both very satisfying and intellectually stimulating. New opportunities for research and development present themselves each day and no single day is exactly like any previous.

## 5 GLOBAL HEALTH

Throughout my entire career, I truly believed that good translational research would come if we had a very active and robust clinical service program. Keeping up with the rapidly developing technology and clinical applications became the biggest challenge but also afforded us many new opportunities. In 2012, our Norris Cotton Cancer Center, one of the NCI designated comprehensive cancer centers, was charged with developing global health initiatives. A meeting with the then cancer center director Dr. Marc Israel led to my being introduced to Linda Kennedy, Associate Director for Strategic Initiatives and Global Oncology. Linda and a group of providers from Dartmouth had been providing basic healthcare needs to a rural village in Honduras. She asked if I would be interested in joining her on the next trip to see if there was a cancer‐based issue that we could study and help resolve. What I expected to be a simple trip to the village turned out to be a tour of the country‘s entire health care system with visits to several villages, outposts, smaller community hospitals, larger city hospitals, and teaching hospitals.

Over the course of the week and speaking with many providers, it became clear that one major cancer‐related problem was the numbers of women developing and dying from cervical cancer. While routine screening was marginal thru Pap smear testing, there were limited resources and only a handful of qualified pathologists in the country to review the slides thereby making turnaround times for results in excess of 6–9 months. As this was to become the focus of our investigation, my team and I developed methods to provide rapid human papillomavirus (HPV) testing in the field via scheduled health fairs and to determine the prevalence of HPV in Honduras.^31^, ^32^, ^33^, ^34^, ^35^ Through multiple health fairs, we defined the prevalence of high‐risk HPV types in regions of Honduras and showed that these were quite different than what we see in the U.S. In addition, we were able to study HPV in cervical cancer tissues from Honduran women and identify which high‐risk HP types were actually progressing from simple infection to cervical cancer. This would have a significant impact on future screening and HPV vaccination programs. What seemed to start out as a simple research question has evolved into multiple studies, interventions and educational opportunities that have impacted thousands of women in that country.

While clinical genomics and molecular diagnostics are no longer considered a discipline in its infancy, some still question the efficacy and viability of this specialty. The evolution of molecular diagnostics and the experiences gained over the last 30 years could not have been better positioned for the response laboratories made to the COVID‐19 pandemic. From the rapid sequencing to identify the virus to the development of reverse transcriptase real time PCR (or just PCR for the lay news) testing, the benefits of those experiences could not have been better underscored. Traditional laboratory testing for antibodies and antigens would not have allowed for the rapid responses and mitigations that were made during this pandemic. Our ability to develop, validate and scale‐up PCR‐based tests for detection of SARS‐CoV‐2 was unprecedented.^37^, ^38^

## 6 SUMMARY

I have been very fortunate to be a part of science and medicine at a time of many new discoveries, changes, and opportunities. Over the years, my laboratory continued to change with the field as the status quo was never an option in this rapidly evolving diagnostic science. My PhD thesis advisor would always say to his students, “finish your PhD, it‘s the right of passage.” At the time, we really weren‘t sure what he meant, but it has been made clear that as PhD scientists there are many doors waiting for you to open. For those contemplating a similar career track as mine, I call your attention to a recent publication that describes the paths you can take.^36^ Other doors may lead to roads less traveled but very fulfilling and enjoyable careers.
